# Long-Period Gratings in Highly Germanium-Doped, Single-Mode Optical Fibers for Sensing Applications

**DOI:** 10.3390/s18051363

**Published:** 2018-04-27

**Authors:** Sebastian Schlangen, Kort Bremer, Yulong Zheng, Sebastian Böhm, Michael Steinke, Felix Wellmann, Jörg Neumann, Bernhard Roth, Ludger Overmeyer

**Affiliations:** 1 Hannover Centre for Optical Technologies, Leibniz Universität Hannover, Nienburger Str. 17, 30167 Hannover, Germany; kort.bremer@hot.uni-hannover.de (K.B.); yulong.zheng@hot.uni-hannover.de (Y.Z.); bernhard.roth@hot.uni-hannover.de (B.R.); ludger.overmeyer@ita.uni-hannover.de (L.O.); 2 Laser Zentrum Hannover e.V., Hollerithallee 8, 30419 Hannover, Germany; s.boehm@lzh.de (S.B.); m.steinke@lzh.de (M.S.); f.wellmann@lzh.de (F.W.); j.neumann@lzh.de (J.N.)

**Keywords:** long-period fiber grating, high NA fiber, amplitude mask, excimer laser, germanium-doped fiber, single-mode fiber

## Abstract

Long-period fiber gratings (LPGs) are well known for their sensitivity to external influences, which make them interesting for a large number of sensing applications. For these applications, fibers with a high numerical aperture (i.e., fibers with highly germanium (Ge)-doped fused silica fiber cores) are more attractive since they are intrinsically photosensitive, as well as less sensitive to bend- and microbend-induced light attenuations. In this work, we introduce a novel method to inscribe LPGs into highly Ge-doped, single-mode fibers. By tapering the optical fiber, and thus, tailoring the effective indices of the core and cladding modes, for the first time, an LPG was inscribed into such fibers using the amplitude mask technique and a KrF excimer laser. Based on this novel method, sensitive LPG-based fiber optic sensors only a few millimeters in length can be incorporated in bend-insensitive fibers for use in various monitoring applications. Moreover, by applying the described inscription method, the LPG spectrum can be influenced and tailored according to the specific demands of a particular application.

## 1. Introduction

Long-period fiber gratings (LPGs) are applied in order to couple light between core and co-propagating cladding modes in single-mode optical fibers. Introduced about two decades ago, LPGs have found many applications due to their characteristics and sensitivity. For example, external perturbations, due to mechanical stress, temperature, or refractive index changes in the surrounding medium, will change the grating period, as well as the effective refractive index of the modes. These effects can be utilized for the development of bend [1], torsion [2], temperature [3], and pressure [4], as well as refractive index sensors [4,5,6], which also find applications as biosensors due to special coatings of the fiber cladding to detect e.g. viruses [7] or bacteria [8]. 

The corresponding coupling wavelength λ_R_ of the LPGs to couple light between the core mode and an individual cladding mode can be calculated as:

(1) $$\lambda_{R}=\Lambda \cdot \left(n_{\mathrm{eff},\mathrm{Cr}}-n_{\mathrm{eff},\mathrm{Cl}}\right),$$

where Λ is the grating period and n_eff,Cr_ and n_eff,Cl_ are the effective refractive indices of the core and cladding mode, respectively. For the fabrication of LPGs, various manufacturing techniques are employed to realize a periodic modulation of the refractive index within the fiber core. Common methods are laser-based, point-by-point inscription systems utilizing, for example, CO_2_ infrared laser radiation operating either in continuous or in pulsed mode [9,10], or ultra-violet (UV) femtosecond laser radiation [11,12]. LPGs can also be realized by inducing mechanical stress or employing other physical modifications such as microbends [13], electric arc-discharges [14,15,16], or ion-implantation [17] whereby the optical spectrum can be optimized by etching processes [18]. One of the simplest and widely used methods for LPG fabrication is the amplitude mask (AM) technique [3,19,20,21,22,23,24,25]. Amplitude masks are periodic arrangements of rectangular and transparent slits that usually have a width of Λ/2, and are separated by the grating period Λ. Furthermore, AMs are usually based on alternating chrome and chrome-less areas on a fused silica glass substrate [19], or metal sheets with rectangular recesses [26]. Compared to the point-by-point fabrication of LPGs, the main advantage of the AM-based manufacturing technique is that a simple, robust, and relatively low-cost optical setup is sufficient, and thus, this manufacturing technique is more suitable for mass production. The UV-induced LPG inscription is only reported for standard telecommunication fibers with numerical apertures (NAs) of 1.2–1.4, such as Fibercore PS1250/1500 (NAs 0.12–0.14), Nufern GF1 (NA 0.13), or Corning SMF-28 (NA 0.14), so far [4,12].

In terms of sensing applications, fibers with a high NA (i.e., fibers with highly Germanium (Ge)-doped fused silica fiber cores) are more attractive since they are less sensitive to bend/microbend-induced light attenuations, and are intrinsically photosensitive, and thus, are suitable for the fabrication of fiber grating structures using UV excimer laser light. The insensitivity to bend/microbend-induced light attenuation is particularly important when integrating optical fibers in functionalized textile structures that are applied for structural health monitoring (SHM) applications, for example [27]. However, when applying single-mode optical fibers with high NAs for use in LPG applications, it follows from Equation (1), that due to the large difference in the effective refractive indices between the core and cladding modes, small grating periods are required for the co-propagating IR coupling in a wavelength range between 1350 to 1600. Since metal masks with small periods are difficult to fabricate, and the durability of chrome masks to high-power UV light exposure is limited, the manufacturing of LPGs in highly Ge-doped, single-mode fibers has not been reported so far.

In this work, we present for the first time a method to inscribe UV-induced LPGs in highly Ge-doped, single-mode optical fibers, which were previously subjected to a tapering process using the AM technique and KrF excimer laser light illumination. For the experiments, the highly Ge-doped, single-mode fiber SM1500 (4.2/125) from Fibercore (Southampton, UK) was applied, which had a high NA of 0.3, and thus, ensured high photosensitivity and an inherent high-bending resistance. Different core and cladding diameters were investigated in order to determine their influence on the required grating period, as well as their impact on the resulting transmission spectrum of the LPGs. Moreover, the sensitivity of the spectrum to refractive index changes of the surrounding medium was exploited. The investigation began with optical simulations of the LPG inscription process, which were subsequently verified by experiments. This was then followed by the measurement of the influence induced by the refractive index change in the surroundings.

## 2. Optical Simulations

The optical simulation of the LPG inscription method was performed in RSoft (Synopsys, Inc., Mountain View, CA, USA) using the GratingMOD toolbox. The following parameters were applied for the Fibercore SM1500 single-mode fiber: refractive index of the fiber core n_Cr_ = 1.47; refractive index of the cladding n_Cl_ = 1.44; and core diameter d_Cr_ = 4 µm. For the LPG, a grating length of L_Gr_ = 8 mm, as well as a refractive index modulation of δn = 10^−3^ within one period of the fiber grating, were assumed. The simulations were performed in a wavelength range between 1350 to 1600 nm. For the simulation model, the refractive indices of the core and cladding were assumed to be constant, and thus, only waveguide dispersion effects were taken into account by the RSoft GratingMOD Toolbox.

### 2.1. Untreated Highly Ge-Doped, Single-Mode Optical Fiber

**Figure 1:**
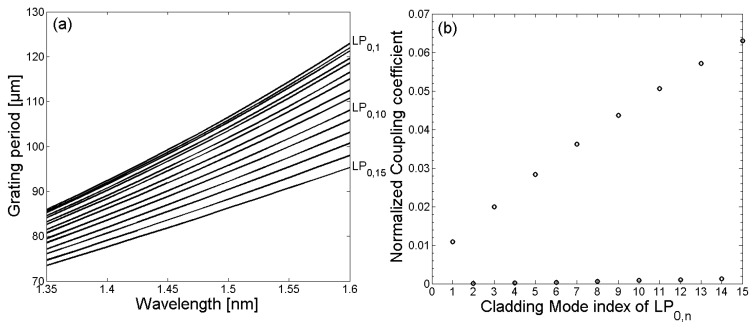
The relationship between grating period Λ and wavelength λ for coupling the core mode into the cladding modes LP_0,1_–LP_0,15_ is shown in (**a**), whereby the grating periods for coupling decrease steadily with increasing mode numbers. The corresponding normalized coupling coefficients are shown in (**b**).

At the beginning of the optical simulation, the required grating periods, as well as the resulting LPG spectrum for the untreated SM1500 single-mode fiber, were determined numerically. In this case, a cladding diameter of d_Cl_ = 125 µm, as well as a refractive index of the surrounding of n_Sr_ = 1 were assumed. The nomenclature of the cladding modes was defined as LP_m,n_, where m = 0 and n = 1, 2, …, 15. In Figure 1a, the calculated grating periods to couple light from the LP_0,1_ core mode to the LP_0,1_–LP_0,15_ cladding modes are illustrated. Moreover, in Figure 1b, the corresponding simulated normalized coupling coefficients are shown.

**Figure 2:**
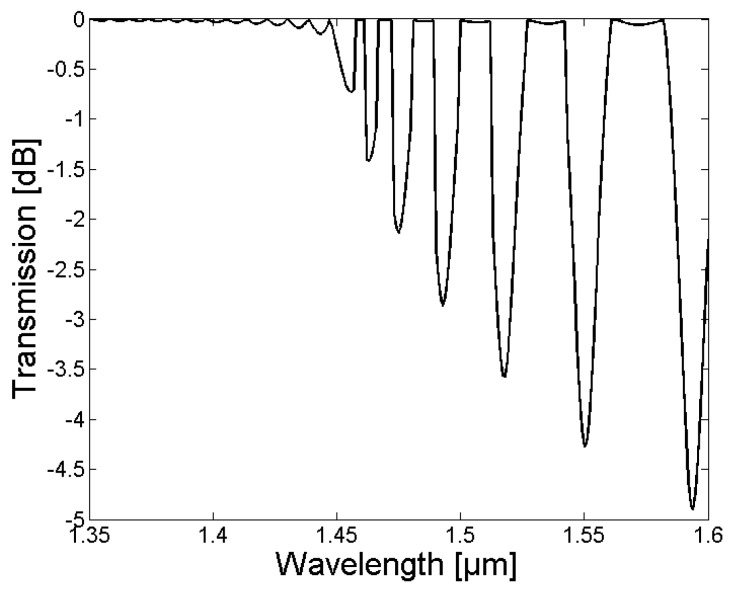
The resulting simulated long-period fiber grating (LPG) spectrum of an untreated SM1500 single-mode fiber with a grating period of 100 µm, a grating length of 8 mm and a refractive index modulation of δn = 1 × 10^−3^.

Due to the relatively small core diameter, which is required in order to provide single-mode operation, resulting in a reduced overlap between the core and cladding mode field distributions, the normalized coupling coefficients between the LP_0,1_ core mode and the LP_0,n_ cladding modes were relatively small. The resulting LPG transmission spectrum for an untreated SM1500 single-mode optical fiber with a grating period of Λ = 100 µm is depicted in Figure 2. According to the calculated grating periods and the coupling coefficients, light was mainly coupled in a wavelength range of 1350 nm to 1600 nm between the LP_01_ core mode and the LP_0,1_, LP_0,3_, LP_0,5_, LP_0,7_, LP_0,9_, LP_0,11_, and LP_0,13_ cladding modes, respectively.

### 2.2. Tapered Highly Ge-Doped, Single-Mode Optical Fiber

From Equation (1), it follows that when the effective refractive index of the core mode is reduced (i.e., when the difference between n_eff,C_ and n_eff,Cl_ decreases), the required grating period of the LPGs increase for a constant coupling wavelength. The effective refractive index of the fundamental core mode depends on the core diameter (d_Cr_), as well as the refractive index difference between the core and the cladding (Δn). Since after the drawing process the refractive index profile of a fiber cannot be tailored, the only alternative is to reduce the core and cladding diameters (e.g., by tapering the fiber) in order to decrease the effective refractive index of the fiber core mode, and to achieve an LPG spectrum in the spectral range between the second and third optical window. This can be reached by applying grating periods of the order of a few hundred microns.

**Figure 3:**
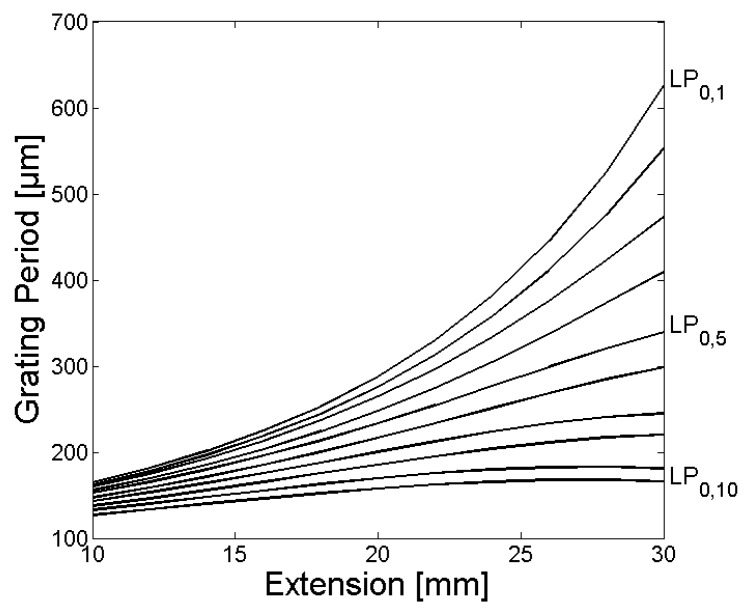
The calculated grating periods to couple light from the fundamental LP_0,1_ core mode to the LP_0,1_–LP_0,10_ cladding modes for the SM1500 fiber with a starting cladding diameter of 125 µm are shown as a function of the fiber extension l, whereby the grating periods for coupling decrease steadily with increasing mode numbers.

**Figure 4:**
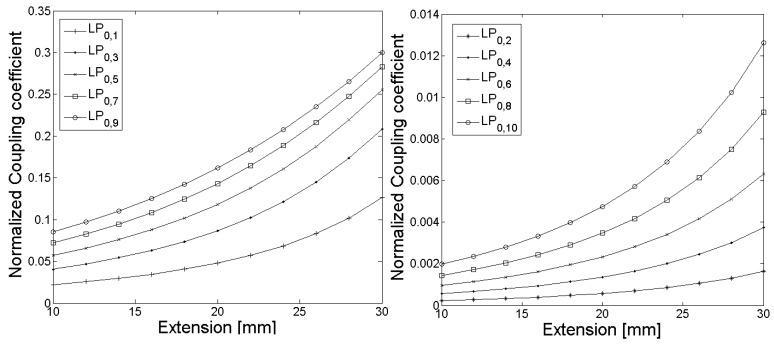
The normalized coupling coefficients as a function of the fiber extension l during the tapering process for coupling of light from the fundamental LP_0,1_ core mode to the LP_0,1_–LP_0,10_ cladding modes of the SM1500 fiber with a starting cladding diameter of 125 µm are shown, whereby modes with odd mode numbers (**left**) have significant larger normalized coupling coefficients compared to the modes with even mode numbers (**right**).

**Figure 5:**
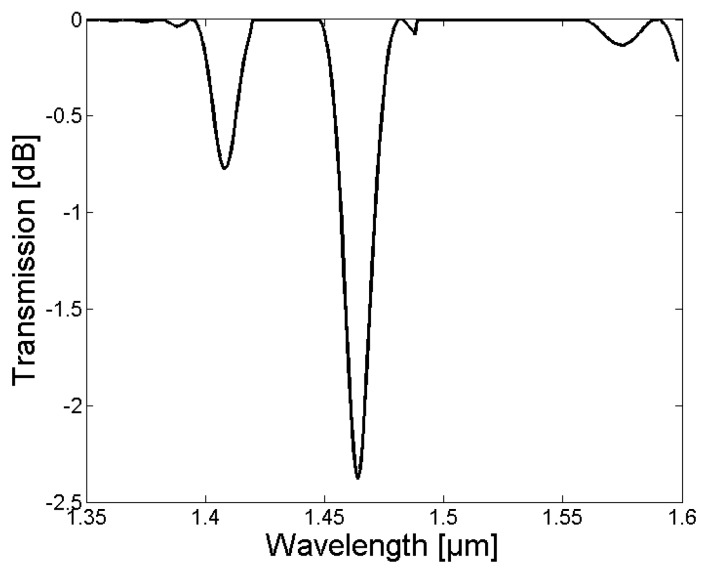
The resulting simulated LPG spectrum in the tapered SM1500 single-mode fiber with a starting cladding diameter of 125 µm, an extension of 28 mm, a grating period of 370 µm, a grating length of 8 mm, and a refractive index change within the grating of δn = 1 × 10^−3^.

**Figure 6:**
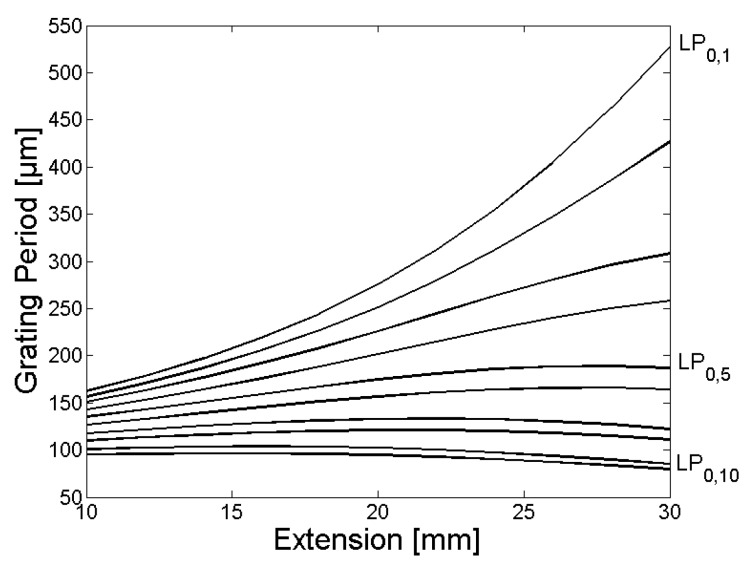
The calculated grating periods to couple light from the fundamental LP_0,1_ core mode to the LP_0,1_–LP_0,10_ cladding modes for the SM1500 fiber with a starting cladding diameter of 80 µm are shown as function of the fiber extension, whereby the grating periods for coupling decrease steadily with increasing mode numbers.

**Figure 7:**
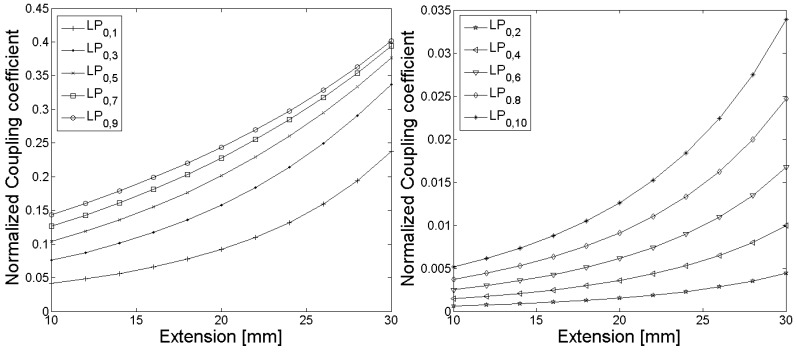
The normalized coupling coefficients as a function of the fiber extension during the tapering process for coupling of light from the fundamental LP_0,1_ core mode to the LP_0,1_–LP_0,10_ cladding modes for the SM1500 fiber with a starting cladding diameter of 80 µm are shown, whereby modes with odd mode numbers (**left**) have significant larger normalized coupling coefficients compared to the modes with even mode numbers (**right**).

**Figure 8:**
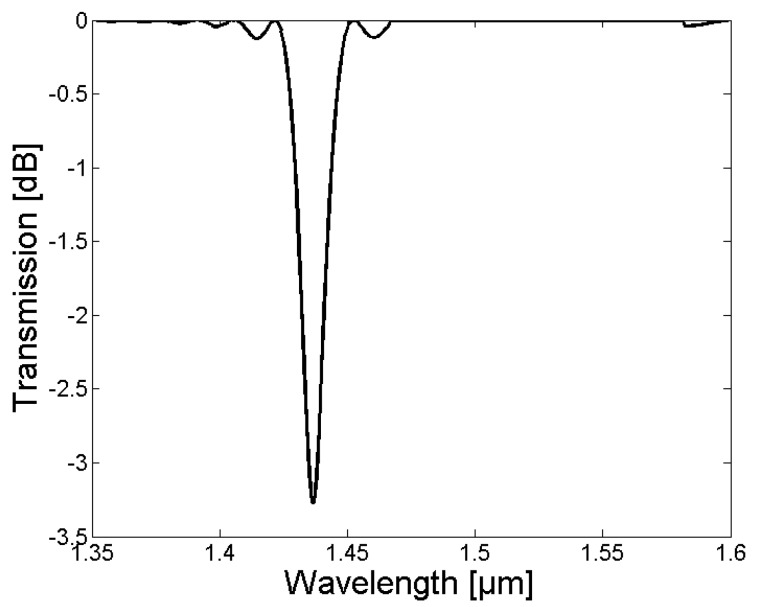
The resulting simulated LPG spectrum in the tapered SM1500 single-mode fiber, with a starting cladding diameter of 80 µm, an extension of 28 mm, a grating period of 370 µm, a grating length of 8 mm, and a refractive index change within the grating of δn = 1 × 10^−3^.

In order to verify this hypothesis, optical simulations of LPGs were performed when the SM1500 single-mode optical fiber was tapered. A parabolic model was applied to model the tapered optical fiber [28]. Based on this model, the diameter of the core d_Cr_ and cladding d_Cl_ can be calculated as [28]:

(2) $$\frac{D_{f}}{D_{i}}=\frac{\frac{3}{2}\left(4\;-\;\frac{l}{\Delta z}\right)}{\left(1\;+\;\frac{l}{\Delta z}\right)\;+\;5{\left[\left(1\;+\;\frac{l}{\Delta z}\right)\left(1\;-\;\frac{l}{5\Delta z}\right)\right]}^{1/2}}\;,$$

where D_i_, D_f_, l, and Δz are the initial and final core and cladding diameter of the fiber before and after tapering, as well as the axial extension length during tapering, and the hot zone of the fiber (i.e., the length of the fiber that was heated during tapering), respectively. For the modelling of the shape of the tapered optical fiber, a hot zone with a length of Δz = 25 mm was assumed for the tapering process. To show how further modification of the LPG spectrum can be achieved by changing the relationship between core and cladding diameter, the optical simulation was performed with cladding diameters of d_Cl_ = 125 µm (Figure 3, Figure 4 and Figure 5) and d_Cl_ = 80 µm (Figure 6, Figure 7 and Figure 8), as the SM1500 fiber is commercially available for both cladding diameters.

In Figure 3 and Figure 6, the calculated grating periods to couple light from the fundamental LP_01_ core mode to the LP_0,1_–LP_0,10_ cladding modes are shown, and the corresponding normalized coupling coefficients as a function of the fiber extension during the tapering process are given in Figure 4 and Figure 7. The LPG transmission spectra for a grating period of Λ = 370 µm and an extension length l = 28 mm are illustrated in Figure 5 (initial cladding diameter of 125 µm) and Figure 8 (initial cladding diameter of80 µm), respectively.

From Figure 3 and Figure 6, it follows that the required grating period can be tailored by tapering the SM1500 single-mode optical fiber to couple light between the fundamental core mode and co-propagating cladding modes. Furthermore, the normalized coupling coefficient between the core and cladding modes, as well as the LPG transmission spectrum, can be optimized (only a single absorption dip appears in a relatively broad spectral range) when reducing the cladding diameter d_Cl_ of the optical fiber prior to the tapering process as one can see in Figure 8.

## 3. Fabrication of LPG in Tapered Highly Ge-Doped, Single-Mode Fiber

**Figure 9:**
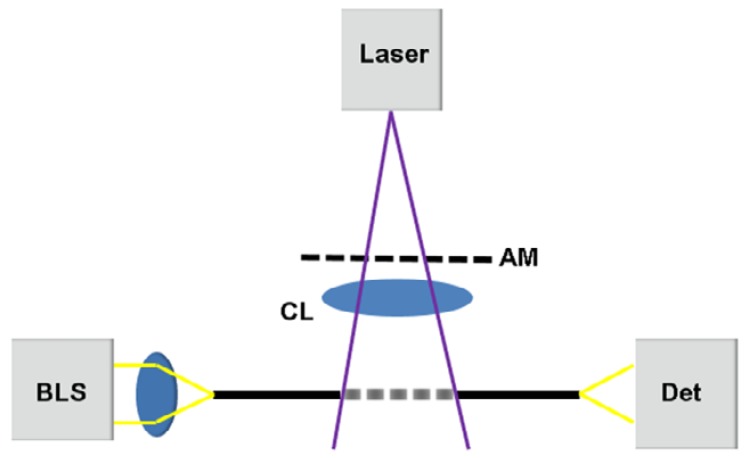
The setup for UV-induced LPG inscription and detection. The UV laser illuminates the amplitude mask (AM), and the generated LPG pattern is focused by a cylindrical lens (CL) in one transversal dimension onto the fiber. During the UV exposure, light is coupled into the fiber using a broadband light source (BLS), and the resulting LPG spectrum is detected at the end of the fiber using an optical spectrum analyzer (OSA) as a detector (Det).

In order to verify the optical simulation, an appropriate fabrication process was developed to inscribe LPGs in the highly Ge-doped, single-mode fiber SM1500. To show how the normalized coupling coefficient between the core and cladding modes, as well as the LPG transmission spectrum can further be optimized so that a single absorption dip appears in a relatively broad spectral range, an initial cladding diameter of 80 µm prior to the tapering process was chosen for the experiments. Since, at this stage, only SM1500 fibers with a cladding diameter of 125 µm were available, the cladding diameter was reduced at the beginning of the fabrication process from d_Cl_ = 125 µm to d_Cl_ = 80 µm (±1.25%) using hydrofluoric acid (40% acid, 60% water). The fiber cladding diameter was etched in a Teflon chamber over a length of 55 mm. Moreover, to obtain a constant etching rate, the temperature was kept constant at 21 °C. Following the etching process, the fiber was tapered. The tapering was done using a hydrogen burner with a melting zone of Δz = 25 mm and extending the fiber by l = 28 mm. After the tapering process, cladding and core diameters of d_Cl_ = 42 µm (± 2.5%) µm and d_Cr_ = 2.1 µm (± 2.5%) µm, respectively, were measured based on a total of 26 fiber tapers using a digital light microscope (Leitz Aristomet (ProMicron, Kirchheim am Neckar, Germany) with Variophot-Tubus and the CCD-Camera AxioCam MRc (Carl Zeiss, Jena, Germany)). The subsequent inscription of the LPG was done by UV light exposure using a KrF excimer laser (Atlex FBG (ATL Lasertechnik GmbH, Wermelskirchen, Germany)) at a wavelength of 248 nm, a pulse energy of 7 mJ, and a repetition rate of 80 Hz. The LPG inscription setup is shown schematically in Figure 9.

As illustrated in Figure 9, the laser beam first illuminates the AM in order to generate the periodic intensity pattern that was required for the LPG inscription. For inscription, chrome masks (Rose Fotomasken Milan, Milos & Eva Rose GbR, Bergisch Gladbach, Germany) were applied. Behind the AM, the spatially modulated beam was focused horizontally using a plan-convex cylindrical lens (UVFS Plano-Convex Cylindrical Lens, f = 10 mm (Thorlabs Inc., Newton, NJ, USA)) to obtain the required intensity density, and the tapered SM1500 fiber was placed in the focal point. The total inscription length of the experimental setup was 8 mm. Since the laser beam was focused to a point behind the AM, the chrome mask was not destroyed during LPG inscription. Moreover, due to the 10 mm gap between the AM and the SM1500 fiber, as well as the vertical divergence of the laser beam, the spatially modulated intensity pattern changed from a perfect rectangular to a sinusoidal-like modulation. Furthermore, to record the LPG transmission spectrum during inscription, a broadband light source (BBS, stabilized fiber-coupled light source SLS201L/M (Thorlabs Inc., Newton, NJ, USA)), as well as an optical spectrum analyzer (OSA, Ando AQ6317B (Ando Electronic SRL, Tokyo, Japan)), were employed and bare fiber adapters were used to connect the fiber under test (i.e., tapered SM1500) to the BBS and OSA. The obtained spectrum was recorded in LabVIEW 2011 SP1 (National Instruments, Austin, TX, USA) and analyzed in MATLAB R2017b (The MathWorks, Inc., Natick, MA, USA).

## 4. Experimental Results of the Inscription Process

**Figure 10:**
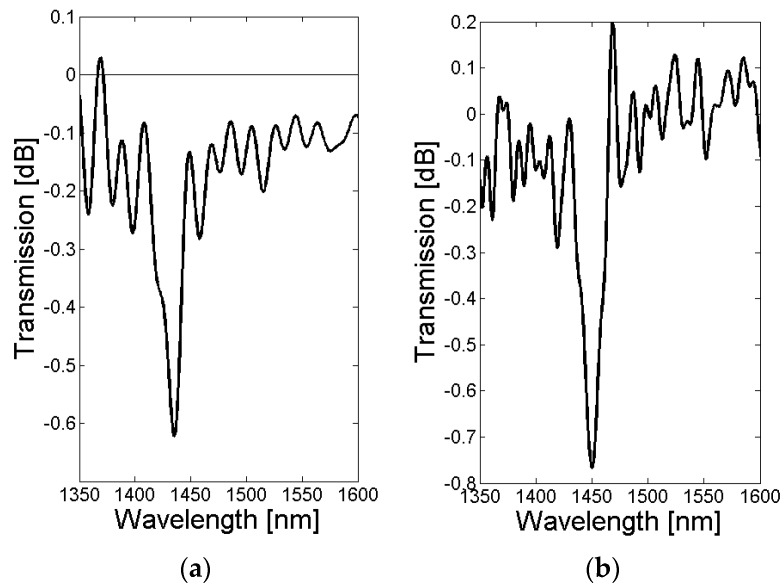
The measured LPG transmission spectra for the tapered SM1500 fiber, with an initial diameter of 81 µm, with an extension of 28 mm, for a grating period of 365 µm and 30 min illumination time (**a**), and a grating period of 370 µm and 45 min illumination time (**b**).

The simulations were experimentally verified by inscribing LPGs in the tapered SM1500 with grating periods of 370 µm and 365 µm, respectively. The recorded LPG transmission spectra for both LPGs are illustrated in Figure 10. The exposure time to UV light from the KrF excimer laser was 30 min and 45 min for the LPGs with a grating period of 365 and 370, respectively.

The transmission spectra of both LPGs show a single absorption dip in the wavelength range from 1408 nm to 1470 nm. The absorption dip was due to the coupling of the LP_0,1_ core mode to the LP_0,1_ cladding mode, and was consistent with the optical simulation in Section 2.2. The difference in the central wavelength of the absorption dip (1435 nm for Λ = 365 µm and 1450 nm for Λ = 370 µm, respectively), shown in Figure 10a,b, can be explained by the different grating periods that were applied for the LPG fabrication.

**Figure 11:**
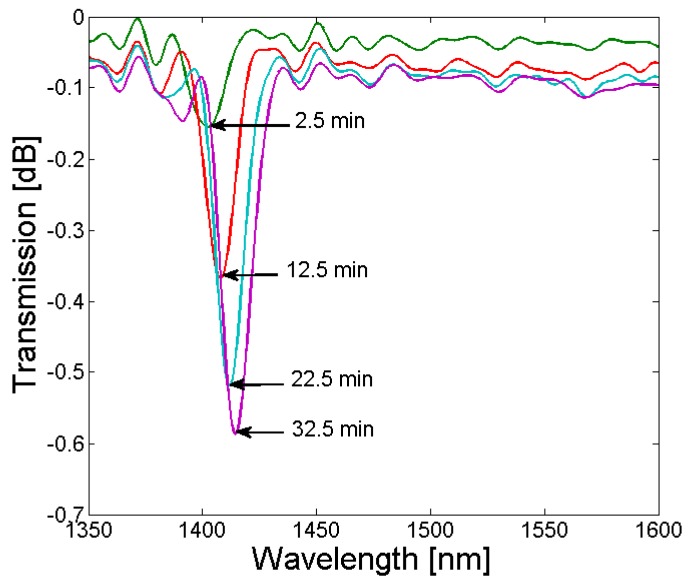
Gradual growth of the absorption dip with increasing UV exposure time, recorded during inscription of an LPG with a grating period of 365 µm in the SM1500 fiber taper, with a starting diameter of 80 µm, and applying a pulse energy of 7 mJ and a repetition rate of 80 Hz.

The strength of the absorption dip depends on the overlap between the core mode and the associated cladding mode field distribution, as well as the refractive index modulation and length of the grating. Since the LPGs shown in Figure 10 were fabricated with different exposure times to KrF excimer laser light, the strength of the absorption dip varies (−0.62 dB for Λ = 365 µm and −0.77 dB for Λ = 370 µm, respectively). In Figure 11, the growth of the absorption dip as a function of KrF exposure time, and thus, refractive index modulation of the fiber core, is illustrated for a grating period of 365 µm and a tapered SM1500 fiber with an extension length of 28 mm and an initial cladding diameter of 80 µm. Currently, the enhancement of the strength of the absorption dip is investigated by loading the tapered SM1500 fibers with hydrogen prior to the grating inscription (to enhance the photosensitivity of the fiber), as well as by replacing the chrome mask with metal sheets (to increase the pulse energy, repetition rate of the laser pulses, as well as the illumination time of the KrF excimer laser without destroying the AM), and increasing the grating length by using a beam expander from 7 mm to a typical length for UV-induced gratings of 10–35 mm [4,10].

The results of the simulated and fabricated LPGs are compared in Table 1. The difference in the simulated and measured results can be explained by inaccuracies in the taper manufacturing process (diameter variations of ±2.5%, as mentioned in Section 3), as well as material dispersion of the SM1500, which was not taken into account during the simulation due to the lack of appropriate material models.

## 5. Response to External Refractive Index Changes

**Figure 12:**
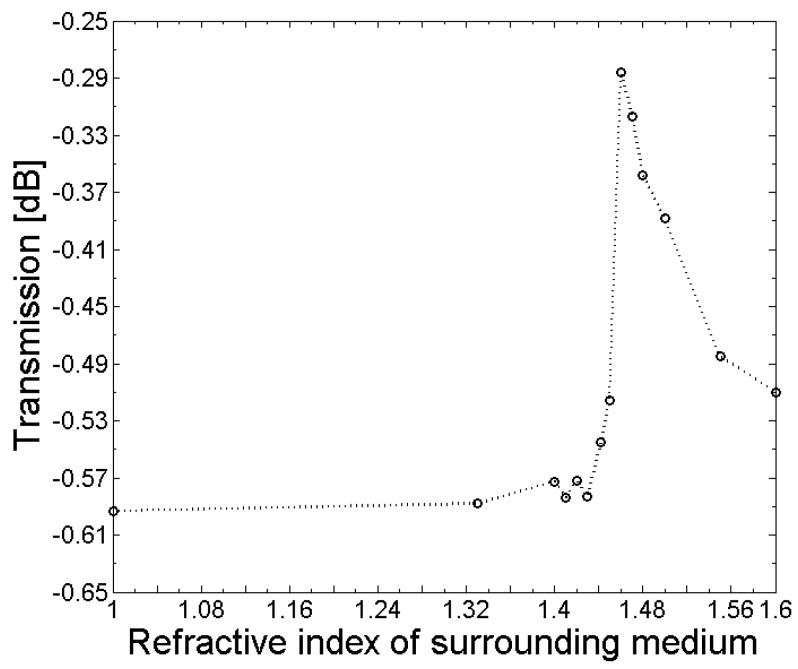
Dependence of the amplitude of the LPG transmission loss dip on the refractive index of the fiber surroundings.

**Figure 13:**
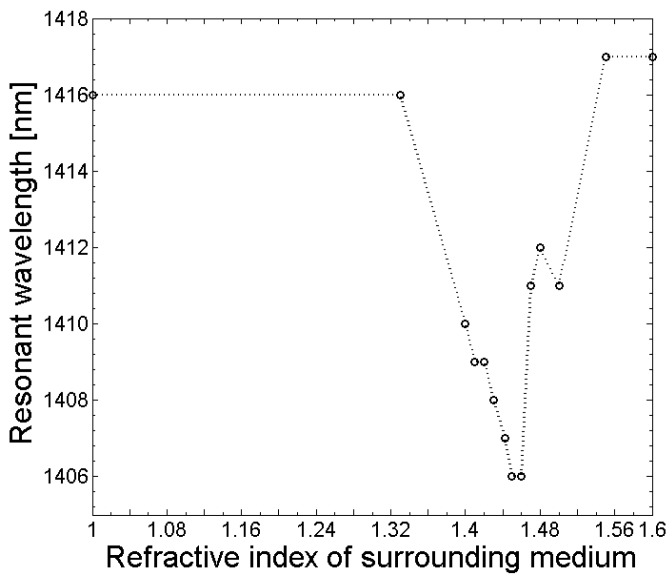
Dependence of the resonant LPG wavelength on the refractive index of the fiber surroundings.

The sensitivity of the fabricated LPGs to refractive index changes in the surroundings was investigated by measuring the transmission spectrum (OSA, Thorlabs SLS201L/M and Ando AQ6317B) and applying different refractive index matching oils (Cargille Laboratories, Inc., Cedar Grove, NJ, USA). After each refractive index measurement, the tapered fiber containing the LPG was carefully cleaned using isopropanol. The responses of the central wavelength and amplitude of the LPG absorption dips are shown in Figure 12 and Figure 13. Moreover, for the experiments, an etched SM1500 fiber with an initial cladding diameter of d_Cl_ = 80 µm was tapered, to a final extension length of 28 mm containing a grating with a grating period of Λ = 365 µm and a length of L_LPG_ = 7 mm. From Figure 12, it follows that in the range between n_s_ = 1 and n_s_ = 1.43, almost no change in the amplitude of the transmission loss dip was measured. However, when the refractive index of the surroundings approached or was equal to the cladding, the refractive index of the amplitude of the transmission dip decreased, and it increased again for refractive index values of the surroundings n_s_ > 1.46. The central wavelength of the LPG absorption dip shifts towards the blue wavelength range with an increasing refractive index of the surrounding medium between n_s_ = 1 and n_s_ = 1.46, as can be seen in Figure 13. For refractive indices of the surrounding n_s_ > 1.46, the central wavelength shifts back towards the red wavelength range, and remains constant for refractive index values n_s_ > 1.5. The obtained response of the amplitude and central wavelength of the LPG absorption dip to different refractive index values of the surrounding medium agrees well with LPGs that have been fabricated using conventional techniques (e.g., [5]).

## 6. Conclusions

In this study, a novel method to inscribe UV-induced LPGs into highly Ge-doped, single-mode fibers was presented. SM1500 fibers from Fibercore were used for inscription of the LPGs, and a single resonance loss dip with up to 0.77 dB attenuation in the wavelength range between 1400–1470 nm was achieved by using grating periods of 365 µm and 370 µm, respectively, and a grating length of 7 mm. Before the LPG was inscribed, the fiber was subject to an etching and tapering process to reduce core and cladding diameter, and thus, to optimize the effective refractive indices of the fundamental core mode and cladding modes, as well as the corresponding coupling coefficients. The fabrication of the LPGs was realized by applying the relatively simple and low-cost amplitude mask technique and a pulsed KrF excimer laser. Since highly Ge-doped, single-mode fibers offer superior features such as low-bending sensitivity and intrinsic photosensitivity compared to other fibers, the novel LPG device could be advantageous in fiber optic sensing applications, such as functionalized textile structures that are applied for SHM applications. The response of the amplitude and central wavelength of the absorption dip of the fabricated LPG spectrum is consistent with LPGs that have been fabricated with conventional fibers. Moreover, the method presented can be beneficial for telecommunication applications or the fabrication of grating assisted mode-selective fiber couplers [29].

## Figures and Tables

**Table 1:** Comparison between the simulated and corresponding experimental LPGs.

Parameter	Simulation	Experiment
initial d_Cr_ (µm)	4	4
initial d_Cl_ (µm)	80	81
Extension (mm)	28	28
Λ (µm)	370	370
L_LPG_ (mm)	8	7
δn	1 × 10^−3^	<1 × 10^−3^
Attenuation (dB)	−3.26	−0.77
λ_central_ (nm)	1436	1449
